# Anxiety, depression, and HRQoL in pediatric microtia patients following ear reconstruction: a cross-sectional study

**DOI:** 10.3389/fpsyt.2025.1625342

**Published:** 2025-11-11

**Authors:** Xinyi Liu, Ying Huang, Lin Yang, Enming Zhang, Jiafei Yang, Liujing Meng, Liping Ma, Zhengzhen Fu, Yanchun Zhou, Weiwei Bian

**Affiliations:** 1 Department of Nursing, Shanghai Ninth People’s Hospital, School of Nursing, Shanghai Jiaotong University, Shanghai, China; 2 Department of Plastic and Reconstructive Surgery, Shanghai Ninth People's Hospital, Shanghai Jiao Tong University School of Medicine, Shanghai, China; 3 University of New South Wales, School of Population Health, Sydney, NSW, Australia

**Keywords:** congenital microtia, anxiety, depression, health-related quality of life, ear reconstruction

## Abstract

**Objective:**

The aim of this study was to evaluate the levels of anxiety, depression, and health-related quality of life (HRQoL) in patients with congenital microtia after ear recontruction surgery and identify influencing factors of HRQoL.

**Method:**

A cross-sectional study was conducted in 152 patients with congenital microtia (aged 8–18 years) who underwent ear reconstruction at a tertiary hospital in Shanghai from April 2023 to September 2024. The assessment tools, including the Hospital Anxiety and Depression Scale, and the Pediatric Quality of Life Inventory Version 4.0 Generic Core Scales, were used to assess symptoms of anxiety, symptoms of depression and HRQoL within 24 hours postoperatively. Multiple linear regression was performed to explore the factors affecting HRQoL.

**Results:**

Postoperatively, 21.7% of patients with congenital microtia reported symptoms of anxiety, and 17.8% reported symptoms of depression. Patients with congenital microtia have significantly lower HRQoL scores than the healthy norm group. Anxiety and depression showed moderate negative correlations with HRQoL score (r = -0.433 and -0.486, respectively, *p* < 0.001). Multivariate analysis showed that residence (95% CI: -6.661, -0.902, *p* = 0.010), surgery type (95% CI: 2.113, 9.496, *p* = 0.002), pain score (95% CI: -1.836, -0.031, *p* = 0.043) and depression score (95% CI: -1.644, -0.318, *p* = 0.004) were significantly associated with HRQoL in patients with congenital microtia after surgery.

**Conclusion:**

Patients with congenital microtia experience significant psychological distress and reduced quality of life after ear reconstruction surgery, with anxiety, depression, pain, and socio-demographic factors playing critical roles. These findings underscore the necessity of an integrated care model that incorporates psychological evaluation, pain management, and personalized support to improve mental health and quality of life in these patients.

## 1 Introduction

Congenital microtia is a common craniofacial condition, applies to anyone born with an underdeveloped ear (1, 2). The global incidence rate is approximately 0.83-17.4 per 10,000 live births, with a prevalence of 3.06 per 10,000 in China, making it the second most common craniofacial condition (3, 4). It predominantly occurs unilaterally, with a higher prevalence on the right side, and is more common in males than females (5).

The auricular underdevelopment is often accompanied by external auditory canal atresia, middle and inner ear deviation, craniofacial asymmetry, and varying degrees of hearing impairment (6). These anatomical abnormalities not only compromise aesthetic appearance and auditory function but also can impact mental health and Quality of Life (QoL) profoundly (7).

A focused investigation into this pediatric population is warranted because their psychosocial experience is fundamentally distinct from that of adults or children with other craniofacial conditions. The impact of microtia coincides with critical developmental periods of identity and body image formation, a challenge different from that of adults with established coping mechanisms. A pediatric focus also ensures an etiologically homogenous cohort of congenital cases, unlike adult populations which can include acquired deformities with different psychological origins. Furthermore, the condition presents a unique dual burden of aesthetic difference and conductive hearing loss, and its standard treatment—multi-stage autologous reconstruction—involves a specific surgical trauma that sets it apart from other craniofacial repairs.

Studies have demonstrated that children and adolescents with congenital microtia can experience a myriad of psychological issues, such as anxiety, depression, social difficulties, sensitivity in interpersonal relationships, hostility, aggression, feelings of loneliness, and diminished self-esteem (7–10). These psychological burdens are compounded by the stigma associated with their appearance, further impacting their QoL (10). Research has reported significantly lower QoL scores among children with congenital microtia compared to their typically developing peers (10).

Ear reconstruction surgery is a common therapeutic strategy to address anatomical defects and improve aesthetic outcomes in patients with congenital microtia. The surgery involves removing the patient’s costal cartilage, carving it into an auricular framework, and implanting it into the affected ear. But at the same time, surgery also brings additional psychological and physiological stress to patients (11, 12). Postoperative pain, prolonged recovery, or unmet expectations regarding surgical results may intensify psychological vulnerability (13).

However, the interrelationships among anxiety, depression status and QoL in patients with congenital microtia remain underexplored. Previous studies have focused predominantly on surgical techniques or pre-operative psychological states (9, 14, 15), leaving gaps in understanding post-operative mental health and QoL. Thus, this study aims to bridge the gap by evaluating the status of anxiety, depression and HRQoL within one day after surgery, and further exploring the influencing factors of HRQoL in the patients. We hypothesized that higher pain, earlier surgical stage, and greater psychological distress would be associated with poorer health-related quality of life (HRQoL). Our findings provide valuable insights into the development of precision nursing interventions that can effectively meet the mental health needs and enhance the overall QoL of patients after ear reconstruction surgery.

## 2 Methods

### 2.1 Subjects

This study was performed on patients who attended Shanghai Ninth People’s Hospital affiliated with Shanghai Jiao Tong University School of Medicine from April 2023 to September 2024. Patients were selected according to the following criteria (1): patients who were diagnosed with congenital microtia through clinical presentation, physical signs, and imaging examinations (16) (2); patients aged between 8 and 18 years who underwent ear reconstruction surgery 3); patients who possessed adequate reading comprehension to complete the survey (4); patients and their guardians agreeing to participate in this study. Exclusion criteria included (1): patients with other severe comorbidities (2); patients with a history of mental illness or cognitive and consciousness impairments (3); patients with communication disorders.

Additionally, 1,583 healthy subjects aged 5–18 years were selected from the Chinese study by Hao et al. (17) as the norm group, comprising 766 males and 817 females. They were recruited via convenient sampling from kindergartens and primary schools in Guangzhou and Shanghai. All students in randomly selected grades were included, except those self-reporting or physician-confirmed acute or chronic illnesses. They completed the same PedsQL 4.0 scale used in our study.

### 2.2 Data collection

Participants were recruited during their hospital stay. The baseline characteristics of the patients included gender, age, educational level, residence, family structure, site of the affected ear, main type and hearing status of the affected ear, and facial asymmetry. Surgery type, pain scores, anxiety, depression, and HRQoL data were collected through questionnaires administered one day after surgery. The selection of demographic and clinical variables was guided by established literature on social determinants of health in pediatric populations and clinical factors known to impact QoL in patients with microtia (8–10). All scales used in this study were self-completed by the patients. To minimize researcher influence, our standard procedure was for the researcher to leave after providing instructions for completing the questionnaires. However, in cases where patients or their family members requested assistance or clarification, the researcher would remain at the bedside. Baseline characteristic information was obtained through interviews with the patients and by reviewing their electronic medical records. The legal guardians were present during these interviews and questionnaire surveys.

The Numeric Rating Scale (NRS) was employed to assess postoperative pain intensity in patients, with scores ranging from 0 to 10 representing different degrees of pain (18). Patients circle a number that most accurately reflects their perceived pain level. The scoring system is stratified as follows: 0–3 points indicate mild pain, 4–6 points indicate moderate pain, and 7–10 points indicate severe pain.

The anxiety and depression levels of patients were assessed using the Hospital Anxiety and Depression Scale (HADS), which was developed by Zigmond and Snaith in 1983 (19). The HADS is a generalized scale with good reliability and validity that is mainly used to screen patients for anxiety and depressive symptoms (20, 21). The HADS comprises two subscales: the Anxiety Subscale (HADS-A) and the Depression Subscale (HADS-D), each containing seven items, resulting in a total of 14 standardized assessment components (20). Each item is scored on a 0–3 point scale, with total subscale scores ranging from 0 to 21. A cutoff score of 8 serves as the clinical threshold for symptom detection, with elevated scores indicating greater severity of anxiety or depressive symptoms (21).

The Pediatric Quality of Life Inventory Version 4.0 (PedsQL 4.0) Generic Core Scales were used to assess the HRQoL in children (22). The PedsQL is a 23-item generic measure that includes four age-specific scales for children aged 2-4, 5-7, 8-12, and 13–18 years. This study used reports from children aged 8 to 12 and adolescents aged 13 to 18, collecting data through self-assessment questionnaires. It assesses four domains: physical health (8 items), emotional functioning (5 items), social functioning (5 items), and school functioning (5 items). Emotional, school, and social functioning can be combined into a psychosocial health domain. Each item is rated on a 5-point Likert scale from 0 to 4, which is subsequently reverse scored and linearly transformed to a scale of 0 to 100 points (0 = 100, 1 = 75, 2 = 50, 3 = 25, 4 = 0). Therefore, a higher converted score indicates a better HRQoL (23). The reliability and validity of the Chinese translations of the PedsQL 4.0 scale have been reported for Chinese children (24). In the current study, the Cronbach’s α for the full scale was 0.894.

### 2.3 Ethical considerations

This study was conducted according to the principles of the Declaration of Helsinki and was approved by the Ethics Committees of Shanghai Ninth People’s Hospital, Shanghai Jiao Tong University School of Medicine (SH9H-2024-T102-1). Written informed consent was obtained from the patient’s parent or legal guardian before collecting the data.

### 2.4 Statistical analysis

Data analysis was conducted using SPSS 26.0 statistical software. Quantitative data that followed a normal distribution were described using the mean ± standard deviation (SD), while non-normally distributed data were presented as the median and interquartile range. Qualitative data were described in terms of frequency, percentage, or constituent ratio (percentage). The independent sample t-test and one-way analysis of variance (ANOVA) were performed for intergroup comparison. Correlation analysis was performed to examine the correlation of HRQoL with anxiety and depression, respectively. Multiple linear stepwise regression analyses were performed with the HRQoL total and domain scores as the dependent variable and the statistically significant variables from the univariate analysis as the independent variables. The independent variables include age, residence, hearing status of the affected ear, pain score, surgery type, anxiety score, and depression score. *R^2^* and ANOVA were used to assess model fit, and fulfillment of assumptions (Durbin Watson, normality of residuals, variance inflation factor and independence) were examined. All tests were performed two-tailed and p values < 0.05 were considered significant.

## 3 Results

### 3.1 Demographic and clinical characteristics

Among the 158 eligible participants, six of them had their surgeries suspended due to colds, while the remaining 152 patients agreed to participate and complete the questionnaires. **Table 1** presents the demographic and clinical characteristics of the participants. For 152 participants, the mean age was 13.53 (± 2.23) years, 107 were males (70.4%) and 45 females (29.6%). Nearly half (48.0%) of participants had an education level of junior high school. More than half (53.3%) of participants lived in the countryside. Most (56.6%) of participants had a nuclear family structure. The majority of participants had congenital absence of the external auditory canal on the affected side, accompanied by impaired hearing and asymmetrical craniofacial development. Regarding the surgery type, 56.6% of participants underwent Stage I ear reconstruction surgery. The average postoperative pain score of the patients was 5.48 (± 2.09) points. Of these participants, 21.7% had postoperative symptoms of anxiety, and 17.8% had postoperative symptoms of depression. More detailed baseline characteristics were listed in **Table 1**.

**Table 1:** Demographic and clinical characteristics of patients with congenital microtia (N = 152).

Characteristics	Number (N)	Ratio (%)	Total score		
			Mean ± SD	*t/F*	*p*
Age			13.53 ± 2.23		
Gender				1.431	0.154
Male	107	70.4	68.22 ± 10.54		
Female	45	29.6	65.43 ± 11.94		
Education level				1.151	0.321
Primary school	38	25.0	67.73 ± 8.53		
Junior high school	73	48.0	66.01 ± 10.08		
Senior high school	41	27.0	69.57 ± 14.11		
Residence				3.831	<0.001
City	71	46.7	70.90 ± 10.10		
Countryside	81	53.3	64.33 ± 10.91		
Family structure				1.632	0.184
Single-parent family	4	2.6	55.98 ± 7.56		
Nuclear family	86	56.6	67.33 ± 10.91		
Extended family	55	36.2	68.44 ± 11.12		
Others	7	4.6	66.61 ± 11.19		
Site of the affected ear				0.297	0.743
Left	50	32.9	68.37 ± 9.97		
Right	97	63.8	66.89 ± 11.59		
Bilateral	5	3.3	67.61 ± 10.69		
Hearing status of the affected ear				4.409	0.014
Normal hearing	11	7.2	76.38 ± 10.84		
Weak hearing	119	78.3	66.99 ± 10.92		
No hearing	22	14.5	65.12 ± 9.82		
External auditory canal				-1.428	0.155
Yes	29	19.1	70.02 ± 10.88		
No	123	80.9	66.78 ± 10.99		
Facial asymmetry				0.393	0.695
Yes	52	34.2	66.91 ± 11.49		
No	100	65.8	67.65 ± 10.80		
Surgery type				-6.479	<0.001
Stage I ear reconstruction surgery	86	56.6	62.90 ± 9.80		
Stage II ear reconstruction surgery	66	43.4	73.25 ± 9.71		
Pain score				19.309	<0.001
Mild pain	30	19.7	76.30 ± 10.38		
Moderate pain	80	52.6	67.07 ± 9.99		
Severe pain	42	27.6	61.67 ± 9.26		
Symptom of anxiety				4.064	<0.001
Yes	33	21.7	60.84 ± 9.78		
No	119	78.3	69.22 ± 10.66		
Symptom of depression				4.626	<0.001
Yes	27	17.8	59.06 ± 10.82		
No	125	82.2	69.20 ± 10.23		

The sum of the percentages is 100% ± 0.1% due to the mathematical characteristics of rounding.

In addition, **Table 1** presents the univariate analysis of factors associated with the total HRQoL score. We found that residence, hearing status of the affected ear, surgery type, pain score, symptoms of anxiety and depression were associated with HRQoL in patients with congenital microtia after ear reconstruction surgery (*p* < 0.001).

### 3.2 HRQoL scores for patients with congenital microtia and healthy adolescents

**Table 2** presents the descriptive statistics determined using independent *t* tests. Compared with healthy adolescents, patients with congenital microtia had significantly lower total HRQoL scores and lower scores in each dimension (*p* < 0.001). Additionally, we calculated Cohen’s d as the effect size for the comparisons (**Table 2**). We found that all Cohen’s *d* values were greater than 0.8, indicating that the statistically significant differences (*p* < 0.001) were not only due to the large sample size but also represented substantial and clinically meaningful differences in HRQoL between patients with congenital microtia and healthy adolescents.

**Table 2:** Comparison of HRQoL scores between patients with congenital microtia and healthy adolescents.

HRQoL scores	NO. of item	Patients with congenital microtia (N = 152)	Healthy adolescents (N = 1583)	*t*	*p*	*Cohen’s d*
		Mean ± SD	Mean ± SD			
Total score	23	67.40 ± 11.00	86.52 ± 9.80	-22.72	<0.001	1.87
Physical health	8	61.13 ± 14.61	87.34 ± 11.97	-25.25	<0.001	2.06
Psychosocial health	15	71.37 ± 12.56	86.08 ± 10.74	-15.88	<0.001	1.30
Emotional functioning	5	68.75 ± 16.31	83.00 ± 14.97	-11.12	<0.001	0.92
Social functioning	5	79.67 ± 18.30	90.04 ± 12.85	-9.11	<0.001	0.69
School functioning	5	65.00 ± 11.02	85.21 ± 13.25	-18.21	<0.001	1.70

### 3.3 HRQoL in patients with congenital microtia stratified by anxiety and depression symptoms

**Table 3** shows that patients with symptoms of anxiety had significantly lower total scores and domain scores of HRQoL compared to those without symptoms of anxiety (*p* < 0.05). Similarly, patients with depressive symptoms showed markedly lower total HRQoL scores and psychosocial health scores (including emotional, social, and school functioning) than those without depressive symptoms (*p* < 0.05). The comparison of physical health scores between patients with and without depressive symptoms did not reach a statistically significant difference (*p* = 0.076), indicating no difference between the two groups.

**Table 3:** Comparison of HRQoL in patients with congenital microtia stratified by anxiety and depression symptoms (N = 152).

Characteristics	Symptoms of anxiety			Symptoms of depression		
	Yes (Mean ± SD)	No (Mean ± SD)	*p*	Yes (Mean ± SD)	No (Mean ± SD)	*p*
Total score	60.84 ± 9.78	69.22 ± 10.66	<0.001	59.06 ± 10.82	69.20 ± 10.23	<0.001
Physical health	55.68 ± 11.99	62.63 ± 14.95	0.015	56.60 ± 11.54	62.10 ± 15.05	0.076
Psychosocial health	63.33 ± 13.5	73.60 ± 11.37	<0.001	60.12 ± 13.48	73.80 ± 10.98	<0.001
Emotional functioning	60.15 ± 15.59	71.13 ± 15.75	0.001	56.48 ± 17.64	71.40 ± 14.79	<0.001
Social functioning	66.97 ± 19.68	83.19 ± 16.31	<0.001	63.89 ± 16.544	83.08 ± 16.87	<0.001
School functioning	59.69 ± 10.23	66.47 ± 10.82	0.002	59.07 ± 12.86	66.28 ± 10.20	0.002

### 3.4 Interrelationships among symptoms of anxiety, depressive symptoms and HRQoL in patients with congenital microtia

The inter-correlation coefficients among symptoms of anxiety, depressive symptoms and HRQoL are shown in **Table 4**. Postoperative symptoms of anxiety were significantly negatively correlated with the total and dimension scores of HRQoL (*r* = -0.433, *r* = -0.218, *r* = -0.464, *r* = -0.422, *r* = -0.430, *r* = -0.314, separately, all *p* < 0.001). A similar result was also observed for postoperative depressive symptoms. With reference to Cohen (25), correlation coefficients of 0.10 to 0.29, 0.30 to 0.49 and 0.50 to 1.0 are interpreted as small, medium and large, respectively. There was a moderately negative correlation between total HRQoL score and both symptoms of anxiety (*r* = -0.433, *p* < 0.001) and symptoms of depression (*r* = -0.486, *p* < 0.001). A high negative correlation was found between psychosocial health and depression symptoms (*r* = -0.511, *p* < 0.001), as well as between social functioning and depressive symptoms (*r* = -0.507, *p* < 0.001).

**Table 4:** Interrelationships among symptoms of anxiety, symptoms of depression and HRQoL in patients with congenital microtia (N = 152).

Variable	Total score	Physical health	Psychosocial health	Emotional functioning	Social functioning	School functioning	Anxiety score	Depression score
Total score	1.000							
Physical health	0.667^**^	1.000						
Psychosocial health	0.891^**^	0.295^**^	1.000					
Emotional functioning	0.809^**^	0.326^**^	0.847^**^	1.000				
Social functioning	0.706^**^	0.152	0.863^**^	0.563^**^	1.000			
School functioning	0.675^**^	0.298^**^	0.719^**^	0.478^**^	0.475^**^	1.000		
Symptoms of anxiety	-0.433^**^	-0.218^**^	-0.464^**^	-0.422^**^	-0.430^**^	-0.314^**^	1.000	
Symptoms of depression	-0.486^**^	-0.269^**^	-0.511^**^	-0.411^**^	-0.507^**^	-0.347^**^	0.791^**^	1.000

^**^p<0.001.

### 3.5 Factors associated with HRQoL

**Table 5** presents the variables that might contribute to decreased HRQoL of patients with congenital microtia. Stepwise multivariate linear regression analysis showed that residence (95% CI: -6.661, -0.902, *p* = 0.010), surgery type (95% CI: 2.113, 9.496, *p* = 0.002), pain score (95% CI: -1.836, -0.031, *p* = 0.043) and depression score (95% CI: -1.644, -0.318, *p* = 0.004) were independently associated with the total HRQoL score of microtia patients. We analyzed the influencing factors of various dimensions of HRQoL and found that age (*p* = 0.001), surgery type (*p* < 0.001) and pain score (*p* = 0.001) were significantly associated with the physical health of microtia patients. Residence (*p* = 0.021), surgery type (*p* = 0.031) and depression score (*p* < 0.001) were significantly associated with the psychosocial health of microtia patients.

**Table 5:** Regression analysis of factors associated with HRQoL in patients with congenital microtia (N = 152).

Variable	*B*	*SE-B*	*β*	*t*	*p*	*95% CI*		*VIF*
						*Low*	*Up*	
Total score ^a^								
Residence ^*^	-3.781	1.457	-0.172	-2.596	0.010	-6.661	-0.902	1.102
Surgery type ^#^	5.804	1.868	0.262	3.108	0.002	2.113	9.496	1.787
Pain score	-0.934	0.457	-0.177	-2.044	0.043	-1.836	-0.031	1.883
Depression score	-0.981	0.335	-0.310	-2.924	0.004	-1.644	-0.318	2.828
Physical health ^b^								
Age	1.452	0.422	0.221	3.440	0.001	0.618	2.287	1.052
Surgery type ^#^	9.771	2.462	0.333	3.968	<0.001	4.904	14.638	1.787
Pain score	-1.962	0.602	-0.280	-3.259	0.001	-3.152	-0.772	1.883
Psychosocial health ^c^								
Residence ^*^	-4.154	1.782	-0.166	-2.331	0.021	-7.675	-0.632	1.084
Surgery type ^#^	3.825	1.760	0.151	2.173	0.031	0.347	7.304	1.044
Depression score	-1.574	0.260	-0.436	-6.063	<0.001	-2.087	-1.061	1.114
Emotional functioning ^d^								
Surgery type ^#^	7.915	2.998	0.241	2.640	0.009	1.991	13.840	1.738
Depression score	-1.542	0.339	-0.329	-4.543	<0.001	-2.213	-0.871	1.093
Social functioning ^e^								
Residence ^*^	-7.250	2.679	-0.198	-2.706	0.008	-12.546	-1.955	1.102
Depression score	-2.113	0.617	-0.402	-3.425	0.001	-3.332	-0.894	2.828
School functioning ^f^								
Age	0.920	0.367	0.186	2.509	0.013	0.196	1.645	1.026
Hearing status of the affected ear ^$^	-5.756	1.842	-0.241	-3.126	0.002	-9.396	-2.117	1.114
Depression score	-0.725	0.251	-0.229	-2.893	0.004	-1.220	-0.230	1.174

a *F* = 15.285, *P* < 0. 001, *R^2^* = 0. 426, adjusted *R^2^* = 0. 398.

b *F* = 15.764, *P* < 0. 001, *R^2^* = 0. 434, adjusted *R^2^* = 0. 406.

c *F* = 22.342, *P* < 0. 001, *R^2^* = 0. 312, adjusted *R^2^* = 0. 298.

d *F* = 15.246, *P* < 0. 001, *R^2^* = 0. 293, adjusted *R^2^* = 0. 274.

e *F* = 8.741, *P* < 0. 001, *R^2^* = 0. 298, adjusted *R^2^* = 0.264.

f *F* = 10.031, *P* < 0. 001, *R^2^* = 0. 214, adjusted *R^2^* = 0. 193.

*B*: unstandardized coefficient; *SE B*: standard error of unstandardized coefficient; *β*: standardized coefficient; *CI:* confidence interval; *VIF:* variance inflation factor.

^*^ Residence: 1 (reference) = City; 2 = Countryside; ^#^ Surgery type: 1 (reference) = Stage I ear reconstruction surgery; 2 = Stage II ear reconstruction surgery; ^$^ Hearing status of the affected ear: 1 (reference) = Normal hearing; 2 = Weak hearing; 3 = No hearing.

Surgery type (*p* = 0.009) and depression score (*p* < 0.001) were significantly associated with the emotional functioning of microtia patients. Residence (*p* = 0.008) and depression score (*p* = 0.001) were significantly associated with the social functioning of microtia patients. Age (*p* = 0.013), hearing status of the affected ear (*p* = 0.002) and depression score (*p* = 0.004) were significantly associated with the school functioning of microtia patients.

## 4 Discussion

This cross-sectional study was designed to investigate the postoperative psychological well-being and HRQoL in pediatric patients with congenital microtia. Our primary research aims were to quantify the prevalence of anxiety and depression and to identify the key demographic and clinical factors that significantly predict HRQoL after ear reconstruction surgery. The findings revealed that a substantial number of patients experience psychological distress and report a significantly lower HRQoL compared to their healthy peers.

Our study found that 21.7% and 17.8% of microtia patients reported postoperative anxiety and depression symptoms, respectively, aligning with previous reports. Although surgical intervention could improve the appearance of the patients to a certain extent (26), a concerning proportion of patients held excessively high expectations for surgery and believed that it could completely restore auricular morphology (27, 28). Concurrently, concerns about suboptimal postoperative recovery frequently arise, often experiencing psychological distress, including anxiety and depression (29). Many studies (30, 31) have reported the psychosocial consequences of microtia patients after ear reconstruction surgery. A study by Horlock et al. (30) involving 62 patients with congenital microtia after auricular reconstruction found that 55% of patients generally experienced anxiety and depression. Another study utilizing the 9-item Patient Health Questionnaire (PHQ-9) and Generalized Anxiety Disorder scale (GAD-7) among 121 perioperative congenital microtia patients under the age of 18 reported that 11.6% of all patients exhibited symptoms of anxiety, and 33.9% of patients exhibited symptoms of depression (31). These studies emphasize the psychosocial challenges faced by patients with craniofacial abnormalities.

This study showed that the total HRQoL score among patients with congenital microtia after surgery was markedly lower compared to healthy adolescents, indicating a substantial reduction in overall QoL. This decrement was observed across all domains of HRQoL, including physical health, emotional functioning, social functioning, and school functioning. The psychosocial health domain, which encompasses emotional, social, and school functioning, was particularly impacted. Notably, we found that hearing status of the deformed ear was significantly associated with the school functioning of patients with congenital microtia. Studies reported that over 90% of patients had varying degrees of hearing impairment, which significantly impacts their learning and daily life (6). These findings align with previous studies highlighting the detrimental effects of congenital microtia on mental health and quality of life (8, 10).

Our results showed that HRQoL scores in patients with anxiety and depression were significantly lower than those in patients without anxiety and depression. There was a moderate negative correlation between symptoms of anxiety, symptoms of depression and total HRQoL scores, emphasizing the critical role of psychological factors in determining QoL outcomes. Healthcare providers should strengthen psychological counseling for patients, clearly explaining the shape and physiological characteristics of the reconstructed ear can only roughly resemble those of a normal ear, and that the cartilage elasticity and microstructures cannot completely match those of a natural ear, which helps improve patient’s acceptance (32). Additionally, psychological support can be provided by establishing online communication groups, organizing in-person patient exchange meetings, and facilitating the sharing of treatment and rehabilitation experiences. These methods can also help alleviate patients’ anxiety and depressive symptoms and improve their QoL.

### 4.1 Associated factors of HRQoL among patient with congenital microtia after ear reconstruction surgery

Multivariate regression analysis revealed that factors such as rural residence, early surgical stages (Stage I), high pain scores, and depressive symptoms were significantly associated with poorer QoL. Rural patients had significantly lower HRQoL than urban patients (*β* = -0.172, *p* = 0.010). This disparity might be related to the inequitable distribution of healthcare resources and insufficient social support. Patients in rural areas faced greater challenges in postoperative follow-up feasibility, limited access to psychological interventions, and potentially heavier financial burdens, which affected recovery outcomes (33). Future interventions should incorporate regional characteristics, such as providing regular psychological assessments through telemedicine or collaborating with community healthcare providers to conduct mental health education, aiming to alleviate the psychological stress of rural patients and improve their HRQoL.

Patients undergoing Stage I surgery demonstrated significantly lower HRQoL compared to those undergoing Stage II surgery (β = 0.262, *p* = 0.002). This difference is likely attributable to the nature of Stage I surgery, which involves harvesting several pieces of costal cartilage for the auricular framework. This procedure results in greater surgical trauma in both thoracic and auricular regions, prolonged recovery, and consequently, more intense postoperative pain (13). Indeed, postoperative pain was a key factor significantly associated with lower HRQoL in our study. Our finding of moderate postoperative pain (mean score 5.48 ± 2.09) is consistent with previous research, such as that by Woo et al. (13), who evaluated pain in microtia patients postoperatively using the visual analogue scale, reporting mean chest pain scores of 5.1 ± 1.6 points at rest and 5.8 ± 2.1 points during coughing, alongside auricular wound pain averaging 4.1 ± 1.4 points. These findings from many studies indicate that patients with microtia experience moderate postoperative pain after ear reconstruction surgery. This underscores that effective management of postoperative pain is a critical component in improving patient HRQoL.

Therefore, multifaceted interventions are crucial, especially following Stage I surgery. We recommend combining proactive pain management with psychological support. Healthcare providers should assist patients in using a thoracic band with compression bandaging at the site of the chest incision to limit thoracic mobility during breathing; they can also instruct patients to press their chest with both hands when coughing. Concurrently, healthcare providers should help patients adopt comfortable positions and recommend distraction techniques such as watching television, listening to music, and socializing (34). For younger patients with low pain tolerance, healthcare providers should offer frequent reassurance and encouragement, administering analgesics as prescribed when necessary. Furthermore, enhancing preoperative education using tools like 3D imaging simulations can help establish realistic expectations. Integrating these strategies should be implemented to facilitate a phased and better-tolerated recovery across treatment stages.

In summary, our study suggests that it is necessary to adopt a comprehensive nursing model and precise interventions, including psychological support, surgical intervention, and pain management, to improve patient’s QoL.

### 4.2 Advantages and limitations

A key strength of this study is its use of validated tools (HADS and PedsQL 4.0), which enhances the reliability of our findings. Furthermore, our use of both univariate and multivariate analysis allowed for a comprehensive exploration of factors associated with HRQoL. Additionally, the comparison with a healthy norm group provided valuable context for interpreting the HRQoL scores of our patient cohort.

This study has several limitations. First, as a cross-sectional design was employed, all data were collected at only one time point. The transient nature of psychological states and QoL metrics in microtia patients may vary over time in intensity and fluctuation depending on different situations. A future longitudinal study to collect data over an extended period is therefore required to evaluate the long-term and dynamic changes in congenital microtia patients’ psychological health and QoL. Second, convenience sampling from a single center may introduce selection bias that limits the generalizability of findings. Future multi-center investigations with expanded sample sizes are warranted to enhance external validity. Third, the children with microtia were 8–18 years old, but the norm group was 5–18 years old, which had a broader age range. The age discrepancy may affect the comparison between the two groups. Lastly, there may be some potential confounding factors such as analgesic regimen or anesthesia type, that were not included in this study. Future investigations should implement more comprehensive covariate selection protocols to strengthen causal inference.

## 5 Conclusions

This study found that in the acute postoperative period, patients with congenital microtia can experience significant psychological distress and a decline in HRQoL after ear reconstruction surgery. Anxiety, depression, pain, and sociodemographic factors are associated with the patient’s HRQoL. These findings suggest the importance of an integrated care model that incorporates early psychological evaluation, pain management, and personalized support to address these immediate postoperative challenges. Future research should employ longitudinal designs to evaluate the efficacy of targeted interventions in improving the long-term mental health and quality of life in patients with congenital microtia after ear reconstruction surgery.

## Data Availability

The original contributions presented in the study are included in the article. Further inquiries can be directed to the corresponding authors.
